# Endometrial cancer with concomitant endometriosis is highly associated with ovarian endometrioid carcinoma: a retrospective cohort study

**DOI:** 10.1186/s12905-022-01917-5

**Published:** 2022-08-05

**Authors:** Aya Ishizaka, Ayumi Taguchi, Tetsushi Tsuruga, Marie Maruyama, Akira Kawata, Yuichiro Miyamoto, Michihiro Tanikawa, Masako Ikemura, Kenbun Sone, Mayuyo Mori, Kaori Koga, Tetsuo Ushiku, Katsutoshi Oda, Yutaka Osuga

**Affiliations:** 1 Department of Obstetrics and Gynecology, Graduate School of Medicine, The University of Tokyo, 7-3-1 Hongo Bunkyo-ku, Tokyo, 113-8655 Japan; 2 Department of Pathology, Graduate School of Medicine, The University of Tokyo, Tokyo, Japan; 3 Department of Integrative Genomics, Graduate School of Medicine, The University of Tokyo, Tokyo, Japan

**Keywords:** Endometriosis, Endometrial cancer, Ovarian cancer, Endometrioid carcinoma

## Abstract

**Background:**

Endometriosis is assumed to be involved in ovarian cancer development, which is called endometriosis-associated ovarian cancer (EAOC). Uterine endometrial cells may be the cell of origin of EAOC. Accumulated carcinogenic changes in the uterine endometrial cells may increase the risk of developing EAOC. To further understand the pathogenesis of EAOCs, we focused on the clinicopathological characteristics of EAOCs in endometrial cancer patients with concomitant endometriosis.

**Methods:**

We retrospectively reviewed 376 patients who were surgically treated for stage I–III endometrial cancer. Clinicopathological characteristics were compared between patients with and without endometriosis. Furthermore, the incidence of simultaneous endometrial and ovarian cancer (SEOC) and the histological characteristics of SEOC were compared between the two groups.

**Results:**

Among 376 patients with endometrial cancer, 51 had concomitant endometriosis. Patients with endometriosis were significantly younger and more frequently had endometrioid G1/G2 tumors than those without endometriosis. The incidence of SEOCs was significantly higher in endometrial cancer patients with endometriosis than those without it (*p* < 0.0001); notably, 12 of 51 endometrial cancer patients with endometriosis (24%) had SEOCs. All of the ovarian cancers in endometrial cancer patients with endometriosis were endometrioid carcinomas. Moreover, even in those without endometriosis, endometrioid carcinoma was the most common histological type of SEOC.

**Conclusion:**

We revealed that endometrial cancer patients with endometriosis had a high probability of SEOC and that endometrioid carcinoma was the most common histological subtype of SEOC regardless of the presence of endometriosis. For patients with endometrial cancer and endometriosis, careful examination of ovarian endometriotic lesions may be important to detect EAOCs.

**Supplementary Information:**

The online version contains supplementary material available at 10.1186/s12905-022-01917-5.

## Background

Endometriosis is defined as the presence of endometrial glands and stroma-like lesions outside the uterine cavity [1]. Although there are several theories on the pathogenesis of endometriosis, the most widely accepted theory is that endometriosis is caused by the regurgitation of the tubal and uterine epithelium through the fallopian tubes into the pelvic cavity [2]. Patients with endometriosis have a higher risk of developing ovarian endometrioid or clear cell carcinoma than those without endometriosis [3–7]. Based on epidemiological findings, endometriosis is considered the origin of ovarian endometrioid and clear cell carcinoma [8–10]. In addition, genomic studies have demonstrated that cancer-related genomic alterations are present in some endometriotic lesions [4, 11–16]. Moreover, a previous report showed that shared genomic alterations were detected in both clear cell carcinoma and endometriotic sites [17–20]. These genomic findings support the hypothesis that endometriosis is the origin of ovarian endometrioid and clear cell carcinoma.

The pathogenesis of endometriosis is considered to involve regurgitation of the uterine endometrium. Recent genomic research has shown that endometriosis-associated ovarian cancers (EAOCs) share a common cell of origin with the uterine endometrium [5, 14]. Because the carcinogenesis of EAOCs is a multi-stage process [14], ovarian endometrioma derived from an endometrium with carcinogenic changes might have a higher chance to develop EAOCs. To further understand the pathogenesis of EAOCs, we focused on the clinicopathological characteristics of EAOCs in endometrial cancer (EC) patients with endometriosis.

## Methods

### Patients

This study was approved by the Research Ethics Committee of the Faculty of Medicine of the University of Tokyo (approval number: 3084-(7), G0683)**.** We retrospectively reviewed 376 patients with stage I–III EC who underwent surgery with curative intent at the University of Tokyo Hospital between 2007 and 2015, as previously described [21]. Clinical data, such as age, body mass index (BMI), and parity were reviewed. The presence of endometriosis and other pathological features, such as histological grade, pathological stage, myometrial invasion, lymphatic vessel invasion, blood vessel invasion, and lymph node metastasis, were obtained from pathological records. Endometriosis was defined as pathologically detected endometriosis in the resected specimen obtained during surgery for EC. EC patients were divided into two groups according to the presence of endometriosis. Patients with adenomyosis without endometriotic lesions outside the uterus were classified into EC patients without endometriosis. Follow-up information was obtained in August 2021. The diagnosis of simultaneous endometrial and ovarian cancer (SEOC) was based on the criteria proposed by Scully and Young [22]. In this study, other than ovarian cancer, one case each of fallopian tube cancer and peritoneal cancer was included in SEOC.

### Statistical analysis

Clinicopathological data were compared by Wilcoxon analysis for continuous variables and by chi-square test for categorical variables in EC patients with and without endometriosis and in EC patients with and without SEOC. In order to investigate the risk factors of SEOC, a multivariate logistic regression analysis was conducted using the following factors as covariates: age, histology of endometrial cancer, myometrial invasion, and presence of endometriosis. Progression-free survival (PFS) and overall survival (OS) were estimated using the Kaplan–Meier method and analyzed using the log-rank test according to the presence of endometriosis. PFS was calculated from the date of surgery to the date of disease progression or death from any cause. OS was calculated from the date of surgery to the date of death from any cause.

All statistical analyses were performed using JMP Pro version 14.1.0 (SAS Institute, Cary, NC, USA). Differences were considered statistically significant at *p* < 0.05.

## Results

### Clinicopathological characteristics of EC patients with endometriosis

Among the 376 patients with EC, 51 had endometriosis. Among them, 21 patients had ovarian endometrioma and the remaining 30 patients had deep endometriosis and/or endometriosis at the surface of the ovaries, the surface of the uterus, and/or peritoneum. The clinicopathological characteristics of patients with EC according to the presence of endometriosis are summarized in Table 1. Patients with endometriosis were significantly younger (*p* = 0.012) and more frequently had endometrioid G1/G2 tumors than those without endometriosis (*p* = 0.036). There were no significant differences in PFS and OS between EC patients with and without endometriosis (Additional file 1: Fig. S1A and B).

**Table 1:** Patients’ characteristics according to the presence of endometriosis

Parameters	Endometriosis + (n = 51)	Endometriosis − (n = 325)	*p*-values
Age at surgery, years, median (range)	52.6 (36–78)	56.8 (23–88)	0.012^a*^
BMI, median (range)	24.8 (17.2– 42.8)	24.2 (14.7–55.7)	0.761^a^
Parity (%)			
0	27 (53)	138 (42)	0.162^b^
≥ 1	24 (47)	187(58)	
T stage (%)			
pT1	44 (86)	256 (79)	0.401^b^
pT2	4 (7.8)	34 (10)	
pT3	3 (5.9)	35 (11)	
Histology of endometrial cancer (%)			
EM G1 or G2	46 (90)	255 (78)	0.036^b*^
EM G3 or others	5 (9.8)	70 (22)	
LVI (%)	9 (18)	73(22)	0.429^b^
BVI (%)	7 (14)	80 (25)	0.071^b^
Myometrial invasion (%)			
< 1/2	37 (73)	205 (63)	0.181^b^
≥ 1/2	14 (27)	120 (37)	
LNM-positive (%)	7 (14)	42 (13)	0.875^b^

*BMI* Body mass index; *LVI* Lymphatic vessel invasion; *BVI* Blood vessel invasion; *EM G1, 2, 3* Endometrioid carcinoma grade 1, 2, 3; *LNM* Lymph node metastasis

^*^Statistically significant; ^a^Wilcoxon analysis; ^b^Chi-square test

### EC patients with endometriosis were frequently accompanied by ovarian carcinoma

**Fig. 1:**
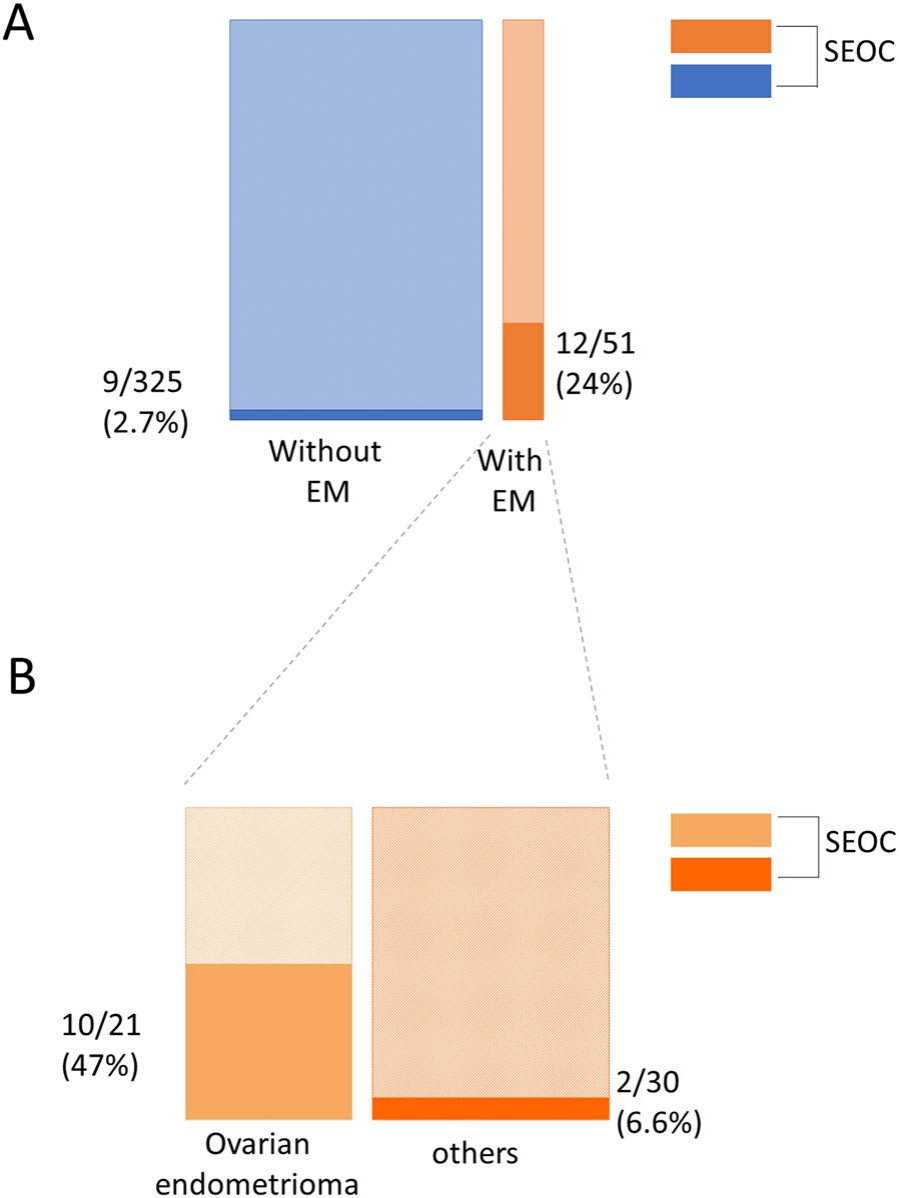
Incidence of simultaneous endometrial and ovarian cancer among endometrial cancer patients. **a** Comparison between endometrial cancer (EC) patients with and without endometriosis. *P* values were obtained using Fisher’s exact test. **b** Comparison between patients with and without ovarian endometriomas among EC patients with endometriosis. EM, endometriosis; SEOC, simultaneous endometrial and ovarian cancer

Out of the 376 total EC patients, SEOCs were found in 21 (5.6%). Patients with SEOC were significantly younger than those without it (*p* = 0.015) and all of them had endometrioid G1/G2 EC (Table 2). When these patients were analyzed based on the presence or absence of endometriosis, the incidence of SEOCs was significantly higher in EC patients with endometriosis than in those without it (Fig. 1A, *p* < 0.0001), with 12 of 51 (24%) EC patients with endometriosis having SEOCs. Furthermore, when endometriosis was analyzed separately by the presence or absence of ovarian endometrioma (regardless of the presence of other types of endometriosis), 10 (47%) EC patients with ovarian endometrioma had SEOCs, while only two (6.6%) without endometrioma had SEOCs (Fig. 1B). We subsequently conducted a multivariate analysis including the following factors as covariates: age, histology of endometrial cancer, myometrial invasion, and presence of endometriosis. We confirmed that the presence of concomitant endometriosis was the independent risk factor for SEOC (Table 3, *p* < 0.0001).

**Table 2:** Patients’ characteristics according to the presence of SEOC

Parameters	EC patients with SEOC (n = 21)	EC patients without SEOC (n = 355)	*p*-values
Age at surgery, years, median (range)	50.6 (39–70)	56.5 (23–88)	0.0189^a*^
BMI, median (range)	23.6 (17.2–34.9)	24.3 (14.8–55.7)	0.411^a^
Parity (%)			
0	11 (52%)	154 (43%)	0.421^b^
≥ 1	10 (48%)	201 (57%)	
T stage (%)			
pT1	16 (76%)	284 (80%)	0.328^b^
pT2	4 (19%)	34 (9.6%)	
pT3	1 (4.7%)	37 (10.4%)	
Histology of endometrial cancer (%)			
EM G1 or G2	21 (100%)	280 (79%)	0.00019^b*^
EM G3 or others	0 (0.0%)	75 (21%)	
LVI (%)	2 (9.5%)	80 (23%)	0.126^b^
BVI (%)	3 (14%)	84 (24%)	0.297^b^
Myometrial invasion (%)			
< 1/2	18 (86%)	224 (63%)	0.016^b*^
≥ 1/2	3 (14%)	131 (36%)	
LNM-positive (%)	1 (4.7%)	48 (13%)	0.193^b^
Presence of endometriosis (%)	12 (57%)	39 (11%)	< 0.0001^b*^

*SEOC* Simultaneous endometrial and ovarian cancer; *EC* Endometrial cancer; *BMI* Body mass index; *LVI* Lymphatic vessel invasion; *BVI* Blood vessel invasion; *EM G1, 2, 3* Endometrioid carcinoma grade 1, 2, 3; *LNM* Lymph node metastasis

^*^Statistically significant; ^a^Wilcoxon analysis; ^b^Chi-square test

**Table 3:** Multivariate analysis for risk of SEOC

	Odds ratio	95% CI	*p*-values
Age at surgery	0.287	0.0116–6.64	0.437
EM G1 or G2	1.70 × 10^8^	Not convergent	0.0207^*^
Myometrial invasion	0.470	0.09–1.64	0.251
Presence of endometriosis	8.98	3.51–23.7	< 0.0001^*^

*CI* Confidence interval; *EM G1, 2, 3* Endometrioid carcinoma grade 1, 2, 3

^*^Statistically significant

### Almost all EAOCs accompanied with ECs were endometrioid carcinoma

**Fig. 2:**
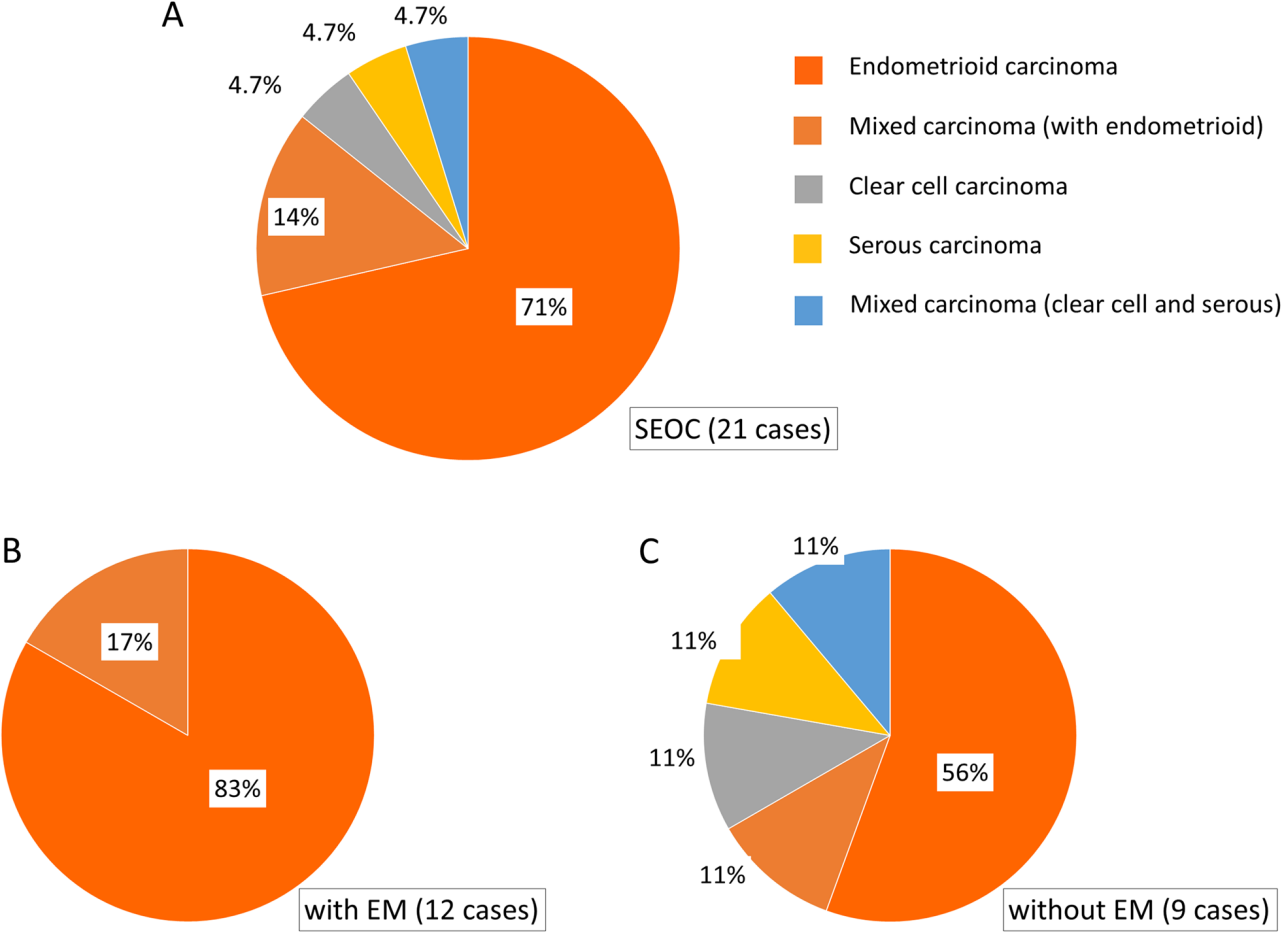
Distribution of ovarian histological types in patients with simultaneous endometrial and ovarian cancer. **a** Total patients. **b** Patients with endometriosis. **c** Patients without endometriosis. SEOC, simultaneous endometrial and ovarian cancer; EM, endometriosis

Among the 21 patients with SEOCs, 18 had endometrioid cancer-related ECs (including three mixed cancers), and the remaining three had clear cell carcinoma (n = 1), serous carcinoma (n = 1), and mixed clear cell and serous carcinoma (n = 1) (Fig. 2A, Additional file 1: Table S1). Generally, EAOCs comprise clear cell and endometrioid ovarian carcinomas, and, in Japan, the incidence of clear cell carcinoma is higher than that of endometrioid carcinoma [23]. However, in our cohort, all of the EAOCs in EC patients with endometriosis were endometrioid carcinoma, and, even in those without endometriosis, endometrioid carcinoma was the most common histological type (Fig. 2B, C, the proportion of endometrioid carcinoma: 100% vs. 66%, *p* = 0.06).

## Discussion

In the current study, we revealed that EC patients who have (concomitant) endometriosis have an increased risk of having SEOCs. In addition, although EAOCs comprise clear cell carcinoma and endometrioid carcinoma, almost all cases of SEOCs that were accompanied by endometriosis were endometrioid carcinoma.

Generally, patients with endometrioid carcinoma G1/G2 have a good prognosis compared to those with endometrioid G3 or other histological types. Although the proportion of endometrioid carcinoma G1/G2 in EC patients with endometriosis was higher than in those without it, there was no difference in prognosis between the two groups. It might be attributable to the high incidence of SEOC in EC patients with endometriosis because ovarian cancer generally has a worse prognosis than EC. In addition, despite the high prevalence of endometrioid carcinoma G1/G2, there is no difference in the frequency of lymph node metastasis. This suggests that patients with endometriosis may have a higher frequency of lymph node metastasis despite endometrioid carcinoma G1/G2.

**Fig. 3:**
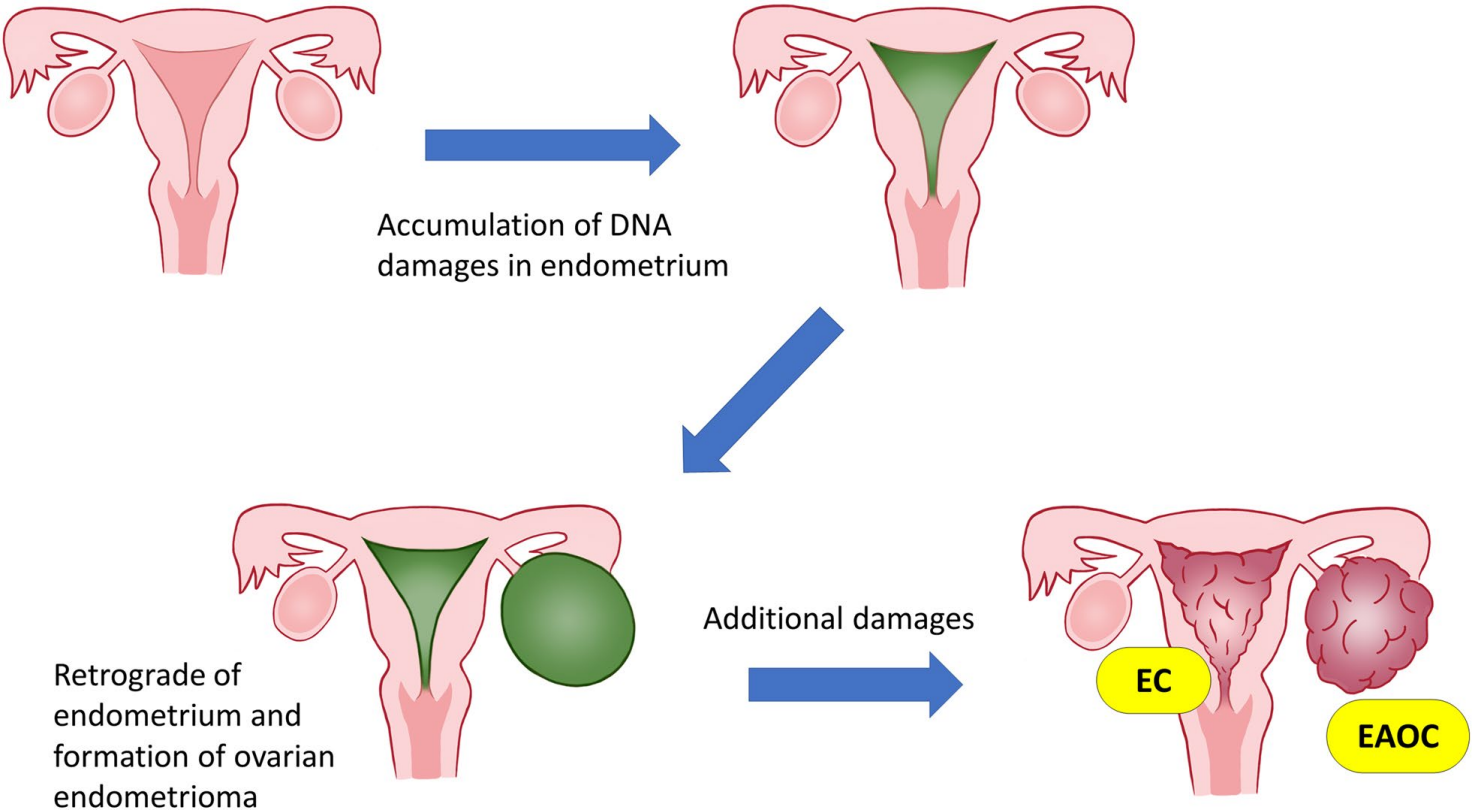
Schema of carcinogenesis of endometriosis-associated ovarian cancer in endometrial cancer patients. EC, endometrial cancer; EAOC, endometriosis-associated ovarian cancer

A prevailing theory for the pathogenesis of endometriosis is the regurgitation of the uterine endometrium [2, 5, 24]. Accumulating evidence suggests that the uterine endometrium is the cell of origin for EAOCs [14]. Recently, in addition to the histopathological approaches, genomic approaches have been gaining attention as the diagnostic methods for SEOCs [25]. The latest genomic approaches based on next-generation sequencing showed clonal commonality between ovarian cancer and endometrial cancer in patients with SEOCs [25–29]. Pathologically diagnosed SEOCs frequently have common oncogene mutations in ovarian and endometrial cancers [25, 26]. These lines of evidence support the hypothesis that endometriosis from the uterine endometrium with carcinogenic changes might be associated with the development of EAOCs and that additional carcinogenic changes might occur after transplantation outside the uterus (Fig. 3). Although EAOC is believed to be derived from endometriosis, the respective carcinogenic mechanisms of clear cell carcinoma and endometrioid carcinoma remain unknown. In the current study, we revealed that almost all SEOCs accompanied by endometriosis were of the endometrioid carcinoma-related type. This finding suggests that ovarian endometrioid carcinoma has a closer relationship with endometriosis from the abnormal uterine endometrium than with ovarian clear cell carcinoma.

It is noteworthy that one-fourth of the EC patients with endometriosis or half of those with ovarian endometrioma were accompanied by EAOC. Considering the high incidence of ovarian cancer in EC patients with endometriosis, treatment strategies should be carefully selected, such as laparotomy, minimally invasive surgeries, and ovary-preserving surgery. In particular, in the case of fertility-sparing therapies for EC patients with endometriosis, it is important to carefully examine endometriotic lesions together with the condition of the uterine endometrium. Atypical endometrial hyperplasia (AEMH) is sometimes accompanied by endometriosis. Ovaries are usually preserved during the treatment of patients with AEMH. However, considering the high probability of ovarian endometrioid carcinoma accompanied by abnormalities in the uterine endometrium, oophorectomy may be considered in patients with endometriosis accompanied by AEMH.

This study has several limitations. First, in the current study, we did not conduct a central pathological review. Therefore, the pathological diagnosis were based on the WHO classifications which were adopted at the time of diagnosis. In addition, the presence of endometriosis was reviewed using pathological reports from electronic medical records. However, some inconspicuous endometriotic lesions may have been overlooked. However, it is still notable that around half of EC patients with ovarian endometrioma have EAOC, and careful examination of the ovaries is necessary for these patients. Second, the diagnosis of SEOC is based on the histopathological diagnosis proposed by Scully and Young [22]. The criterion contains 12 features of SEOC, for example, there is no myometrial invasion or lymphovascular space invasion in endometrial cancer, and there is no ovarian cancer dissemination in the abdominal cavity. However, various investigators using these criteria sometimes arrive at different conclusions. In addition, considering the shared clonality between ovarian cancer and endometrial cancer in patients with SEOC, it might be difficult to accurately distinguish ovarian metastasis from SEOC, even with genomic analysis. To elucidate the pathogenesis of EAOCs, further research is warranted to investigate the molecular changes in pathologically normal uterine endometrium of patients with EAOCs. Third, there are ovarian endometrioid carcinomas that are not associated with ovarian endometriosis [30, 31]; therefore, the hypothesis that ovarian endometrioid carcinoma is arising from endometriosis from the abnormal uterine endometrium might be applied to only a part of ovarian endometrioid carcinomas. Further pathological and genomic analyses are needed to classify the pathogenesis of ovarian endometrioid carcinoma. Lastly, in the current study, we did not investigate the association between adenomyosis and EC. Because mutations in cancer-related genes are detected in adenomyosis [32], the presence of adenomyosis might affect the incidence of EC. Further research is warranted to elucidate the significance of adenomyosis in the development of EC.

## Conclusion

In this study, we revealed that EC patients with endometriosis had a high probability of accompanying ovarian cancer and that ovarian endometrioid carcinoma was the most common histological type. For patients with endometriosis accompanied by abnormalities in the uterine endometrium, careful examination of endometriotic lesions may be important for the early detection of EAOCs.

## Supplementary Information

**Additional file 1: Fig. S1.** Kaplan-Meier survival analysis of endometrial cancer patients. **A** Progression-free survival rate according to the presence of concomitant endometriosis. **B** Overall survival rate according to the presence of concomitant endometriosis Log-rank test was used for *p*-values. **Table S1.** Pathological characteristics of SEOC cases.

## Data Availability

All data generated or analyzed during this study are available from the corresponding author, upon reasonable request.

## Abbreviations

EAOC: Endometriosis-associated ovarian cancers
EC: Endometrial cancer
SEOC: Simultaneous endometrial and ovarian cancer
PFS: Progression-free survival
OS: Overall survival
AEMH: Atypical endometrial hyperplasia
