# Supplementary material for: Endometrial cancer with concomitant endometriosis is highly associated with ovarian endometrioid carcinoma: a retrospective cohort study
Source: BMC Womens Health. 2022 Aug 5;22:332. doi: 10.1186/s12905-022-01917-5 (PMC9354371; doi:10.1186/s12905-022-01917-5)
Supplement: Supplementary file 1 — Additional file 1: Fig. S1. Kaplan-Meier survival analysis of endometrial cancer patients. A Progression-free survival rate according to the presence of concomitant endometriosis. B Overall survival rate according to the presence of concomitant endometriosis Log-rank test was used for p-values. Table S1. Pathological characteristics of SEOC cases. [file 12905_2022_1917_MOESM1_ESM.pdf]

Supplementary Figure

Fig. S1

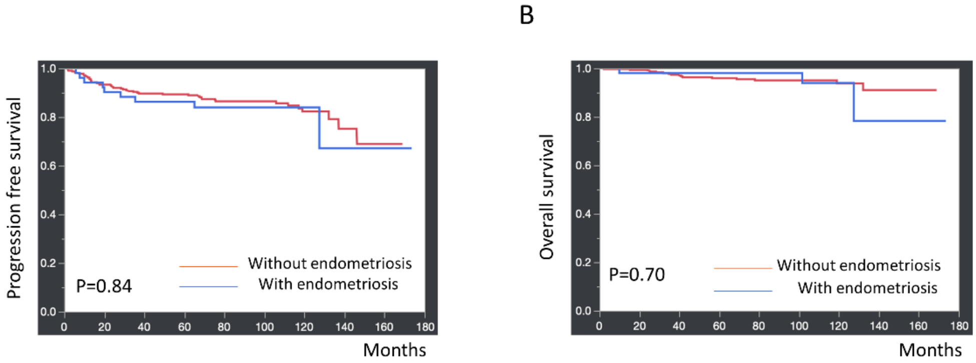

Figure S1 Kaplan-Meier survival analysis of endometrial cancer patients

A. Progression-free survival rate according to the presence of concomitant endometriosis

B. Overall survival rate according to the presence of concomitant endometriosis

Log-rank test was used for *P*-values

Supplementary Table

Table. S1 Pathological characteristics of SEOC cases

Case	Age	Presence of endometriosis	Histology of endometrial cancer	Histology of ovarian cancer
Case 1	39	Positive	EM G1	Endometrioid carcinoma
Case 2	40	Positive	EM G1	Endometrioid carcinoma
Case 3	43	Positive	EM G1	Endometrioid carcinoma
Case 4	44	Positive	EM G1	Endometrioid carcinoma
Case 5	44	Positive	EM G1	Mixed (clear cell + endometrioid)
Case 6	55	Positive	EM G1	Endometrioid carcinoma
Case 7	50	Positive	EM G2	Endometrioid carcinoma
Case 8	48	Positive	EM G1	Endometrioid carcinoma
Case 9	55	Positive	EM G2	Endometrioid carcinoma
Case 10	54	Positive	EM G1	Endometrioid carcinoma
Case 11	50	Positive	EM G1	Mixed (clear cell + endometrioid)
Case 12	44	Positive	EM G1	Endometrioid carcinoma
Case 13	42	Negative	EM G1	Endometrioid carcinoma
Case 14	42	Negative	EM G1	Endometrioid carcinoma
Case 15	49	Negative	EM G1	Clear cell carcinoma
Case 16	53	Negative	EM G1	Endometrioid carcinoma
Case 17	59	Negative	EM G1	Endometrioid carcinoma
Case 18	60	Negative	EM G1	Endometrioid carcinoma
Case 19	67	Negative	EM G1	Serous carcinoma
Case 20	70	Negative	EM G1	Endometrioid carcinoma and seromucinous carcinoma
Case 21	56	Negative	EM G1	Mixed (clear cell + serous)

SEOC: simultaneous endometrial and ovarian cancer; EM G1, 2: endometrioid carcinoma grade 1, 2.
